# Factors associated with the productive longevity of sows in commercial breeding herds

**DOI:** 10.5194/aab-67-297-2024

**Published:** 2024-07-02

**Authors:** Gerardo Ordaz, Manuel López, Rosa E. Pérez, Gerardo Mariscal, Ruy Ortiz

**Affiliations:** 1 Centro Nacional de Investigación Disciplinaria en Fisiología y Mejoramiento Animal, INIFAP, Querétaro, Mexico; 2 Facultad de Medicina Veterinaria y Zootecnia, Universidad Michoacana de San Nicolás de Hidalgo, Michoacán, Mexico; 3 Facultad de Químico Farmacobiología, Universidad Michoacana de San Nicolás de Hidalgo, Michoacán, Mexico

## Abstract

Maximizing sows' productive longevity (PL) represents a significant challenge faced by the swine industry, as the growing increase in the removal rate of sows, mainly young sows, directly impacts the system's economy. In addition, there are ethical concerns associated with animal welfare issues due to the low PL of sows. The objective of this study was to identify and evaluate the risk factors influencing the removal of sows from commercial swine production systems. The variable of interest was the PL of sows. The PL was modeled using Cox regression analysis to identify the factors that affected this variable. The factor with the greatest contribution to PL was sow type (ST), followed by the return to estrus percentage (REP), herd size (HS), season, lactation duration, weaning–estrus interval (WEI), piglets born alive, mummy percentage, and total piglets born. The removal risk was higher for hyperprolific sows than for normal sows. According to the nonproductive day (NPD) variable (an indicator that considers REP and WEI in its calculation), sows with more than 60 nonproductive days per year are at higher risk of elimination. The risk of removal was higher for sows from large herds than for sows from medium or small herds. The PL of sows within a herd is determined by the type of sow and the sows' association with environmental disturbances, including climatic factors (artificial climate control), management practices (human resources), and economic resources (size and infrastructure).

## 1 Introduction

Globalization is the global integration of economic, political, technological, and sociocultural aspects, and swine production systems (SPSs) are not exempt from this process. Owing to globalization, there is greater interconnectivity with respect to innovation and production processes among SPSs (Kotarev, 2019). However, global interconnectivity between SPSs is not always reflected in production benefits, as the geographic region and the specific context of each SPS are factors to consider to guarantee productive efficiency (Dolman et al., 2012; Kuncová et al., 2016). Therefore, knowing the differences between countries (and even within a country) with respect to social, economic, and environmental issues; infrastructure; herd health; genetics; feeding programs; and management practices could lead to greater productive efficiency, as problems can be addressed in a specific way according to the conditions of the SPS in question (Engblom et al., 2007; Tani et al., 2018; Koketsu and Iida, 2020). Conditions may even be specific from a molecular point of view (proteome, metabolome, and microbiome of the animal) according to the previously described particularities of each SPS (Goldansaz et al., 2017; He et al., 2023).

During the last few decades, SPSs have used the number of weaned piglets per sow per year as an indicator that determines the productive efficiency of these systems (Koketsu et al., 2017; Koketsu and Iida, 2020). However, this variable is only related to herd productivity in the short term. Currently, the criterion for evaluating the productive efficiency of SPSs is the productive longevity of sows (Patterson and Foxcroft, 2019; Koketsu and Iida, 2020). Therefore, the sow retention rate has become a key indicator of the economic efficiency of modern pig breeding (Gruhot et al., 2017). The productive longevity of a sow is defined as the farrowing number at the time of sow removal from the herd (Engblom et al., 2007; Koketsu and Iida, 2020), and its importance lies in the number of piglets that a sow can produce in its productive life within the herd. With the current genotypes, it is estimated that sows produce more than 70 offspring throughout their productive lifetime (Lucia et al., 2000; Patterson and Foxcroft, 2019). However, within SPSs, it is common to find sows that only produce between 30 and 40 offspring throughout their productive life; this lower productivity is associated with high sow removal rates from herds (Rodriguez-Zas et al., 2003). Hence, it is important to determine the factors affecting sow removal from herds and their effects on increasing the productive longevity of sows, which is desirable.

To establish the factors associated with the productive longevity of sows within a herd, survival analysis is a useful method to weigh this variable (Szabó and Dákay, 2009), as the association between risk factors and involuntary removal can be evaluated with respect to its effect on the productive life within the herd, instead of a reductionist analysis that describes the productive longevity of the sow in terms of removal (Engblom et al., 2007). In survival analysis, the risk of removal is modeled instead of the productive longevity of the sow. Risk is linked to uncertainty about future events; therefore, it is impossible to eliminate it without considering it, particularly if it is associated with economic risk (Mészáros et al., 2013). In this study, risk represents the probability that an animal will be removed at a given time, given that it is still present in the herd. In this sense, modeling the risk of involuntary removal and not longevity makes it possible to use data from animals that have not yet been removed from the herd as well as those that have already been removed (Engblom et al., 2007). This study aimed to identify and evaluate the risk factors inherent to sows at the farm level that influence their removal from commercial SPSs.

## 2 Materials and methods

As the data evaluated were obtained from electronic records maintained by SPSs, approval from an ethics committee for the care and use of animals was not required.

### 2.1 Herds

This study used data from eight commercial SPSs located in southern central Mexico. The SPSs were selected based on their ability to obtain reliable data. All SPSs captured the reproductive and productive information of the herd using the PigKnows™ software (PigKnows LLC, Greeley, Colorado, USA). The mean size of the evaluated breeding herds was 3327 sows, ranging from 1792 to 7312 sows: SPS-1, 1792 normal sows (NSs); SPS-2, 1943 hyperprolific sows (HPSs); SPS-3, 1834 NSs; SPS-4, 1823 HPSs; SPS-5, 3291 NSs; SPS-6, 3102 HPSs; SPS-7, 5525 NSs; and SPS-8, 7312 HPSs. The number of workers per sow varied by system, with 1 worker per 150 sows for systems with <3000 sows, 1 worker per 200 sows for systems with 3000–5000 sows, and 1 worker per 300 sows for systems with >5000 sows. This disparity with respect to personnel requirements was particularly evident in cleaning and feeding difficulties.

The sows were fed diets based on corn and soybean meal according to their productive stage. Pregnant sows were fed 3.4 $\mathrm{Mcal}\;\mathrm{ME}\;{\mathrm{kg}}^{-1}$ (where ME denotes metabolizable energy), 12.5 % crude protein (CP), and 1.2 $g\;{\mathrm{kg}}^{-1}$ digestible lysine. Lactating sows were fed 3.4 $\mathrm{Mcal}\;\mathrm{ME}\;{\mathrm{kg}}^{-1}$ , 17.5 % CP, and 2.5 $g\;{\mathrm{kg}}^{-1}$ digestible lysine. Vitamins and minerals were added to meet or exceed the recommended requirements of NRC (2012). In all SPSs, feeding during pregnancy was semiautomated and divided into two portions given at 08:00 and 16:00 LT (local time, UTC -6 ). During lactation, feeding was manual, stimulating the feed intake of sows four times a day (08:00, 12:00, 16:00, and 20:00 LT); the feed intake was ad libitum. Regarding the sows' farrowing synchronization, SPS-2 and SPS-4 used oral progestin from day 111 to 113 of gestation. In all other systems, 24 h after farrowing began in the maternity wards, prostaglandins were applied to the vulva of the sows that had not yet given birth to homogenize the age of the litters. In all SPSs, oxytocin or carbetocin was applied to the sows to accelerate uterine contractions as needed. The lactation duration ranged from 12.7 to 27.1 d; for analysis purposes, this variable was classified into two levels, lactations of ≤21 d and lactations of >21 d. After weaning, the sows were housed in groups of 20 ± 5 animals each. Estrus was monitored twice a day with the help of the boars in all SPSs. The eight SPSs used artificial insemination and double or triple insemination of sows during estrus. Pregnancy was monitored in all herds using different types of ultrasound equipment. In five SPSs, the criterion considered for the removal of a sow from the herd was two consecutive returns to estrus, whereas three SPSs used the criteria of three or more successive returns to estrus. The replacement sows in all SPSs were the product of internal multiplication programs. The base sow breeding populations originated from different genetics companies in the United States (US) or European Union (EU), and the systems were later repopulated using the crossover schemes established by each genetics company.

### 2.2 Data

Data from the eight SPSs included 26 622 sows recorded from January 2019 to December 2021. The data comprised the reproductive and productive indicators of the sows (Table 1).

**Table 1:** Reproductive and productive indicators according to the type of sow: hyperprolific vs. prolific.

	Normal sows				Hyperprolific sows			
Indicator	Mean (SEM)	Min	Max	CV	Mean (SEM)	Min	Max	CV
Performance gestation–farrowing–lactation								
Total piglets born ${}^{*}$	13.4 (0.06)	8.0	18.3	7.6	14.3 (0.08)	10.1	19.0	7.7
Piglets born alive ${}^{*}$	11.5 (0.07)	6.4	14.8	10.1	12.0 (0.09)	8.1	18.5	10.8
Stillborn piglets, %	10.1 (0.33)	1.3	27.8	52.1	9.8 (0.39)	1.0	34.1	55.2
Mummies, % ${}^{*}$	6.9 (0.31)	0.0	38.7	71.5	7.8 (0.45)	0.0	40.7	81.9
Pre-weaning mortality, %	13.2 (0.42)	3.1	43.9	48.9	13.4 (0.55)	3.5	52.7	56.8
WPs	10.1 (0.33)	5.7	12.5	11.0	10.5 (0.12)	6.6	14.0	15.9
Lactation duration, d ${}^{*}$	20.7 (0.12)	16.1	25.0	9.2	20.9 (0.15)	12.7	27.1	9.8
Post-weaning performance								
Weaning–estrus interval, d ${}^{*}$	7.0 (0.20)	1.0	28.4	43.8	8.1 (0.25)	3.4	22.0	44.6
Return to estrus, % ${}^{*}$	14.2 (0.67)	11.2	51.1	54.1	15.4 (0.51)	7.1	55.6	57.2
Nonproductive days per sow per year	46.9 (0.88)	20.0	92.3	30.8	36.9 (0.71)	12.4	58.5	27.7
Performance during the sow's productive life								
Farrows per sow per year	2.4 (0.01)	2.0	2.6	4.1	2.4 (0.01)	2.2	2.6	3.0
WPs per year	23.6 (0.15)	12.1	28.8	11.1	25.1 (0.19)	13.1	31.4	10.9
WPs per sow during its productive lifetime	39.3 (0.38)	12.1	60.3	15.6	35.3 (0.82)	7.0	72.9	32.1
Farrowing number upon removal	3.9 (0.03)	2.0	5.2	13.3	3.4 (0.06)	1.0	5.1	27.4

The abbreviations used in the table are as follows: SEM – standard error of the mean; CV – coefficient of variation; WPs – weaned piglets. ${}^{*}$ Indicators used for Cox multivariate analysis according to statistical significance ( P<0.05 ).

In addition, the sow type (normal or hyperprolific) and herd size (HS) – small ( <3000 sows), medium (3000–5000 sows), or large ( >5000 sows) – were considered variable factors. The sow type (ST) was established according to Solà-Oriol and Gasa (2017). Sows of the Yorkshire, Large White, and Landrace genotypes as well as crosses between these breeds with an average litter size history (retrospective analysis of the SPSs) of ≈13 piglets were considered normal sows. For hyperprolific sows, animals belonging to commercial genetic lines with the capacity to produce ≥15 piglets per litter were considered. The productive life of the sows was defined according to two indicators: (i) the farrowing number upon removal and (ii) the number of days between the first farrow and the removal or completion of the collection of sow data. This is because it has been reported that the farrowing number at which the sow is removed is not an accurate way to monitor productive longevity, as the farrowing number at the time of removal does not consider the number of days that the sow was within the herd, which is an indicator that can vary between herds for sows of the same parity. Therefore, the number of days of life of sows within a herd should be used to measure productive longevity (Koketsu and Iida, 2020).

### 2.3 Statistical analysis

All statistical analyses were performed using SAS^®^ version 9.4 (SAS Institute Inc., Cary, North Carolina, USA) and IBM SPSS^®^ version 28.0.1.0 (IBM Corp., Armonk, NY, USA).

A survival analysis methodology was used to identify and evaluate the impact of the indicators that influenced the removal of sows from the SPSs. The risk of removal during the productive life of the sows was analyzed using the Weibull model (Mészáros et al., 2013):

1 $$h(t)=h_{0}(t)\mathrm{exp}[x(t{)}^{\prime }\beta ].$$

Here, $h_{0}(t)$ is the reference hazard function $\lambda p(\lambda t{)}^{p-1}$ , which follows a Weibull distribution with scale parameter λ and shape parameter p ; t is time, expressed as the farrowing number or days of life in the herd; and β contains the (possibly time-dependent) covariates that affect hazard with the corresponding design vector $x^{\prime }(t)$ , where ρ<1 indicates that the risk decreases over time and ρ>1 indicates that the risk increases over time.

**Figure 1:**
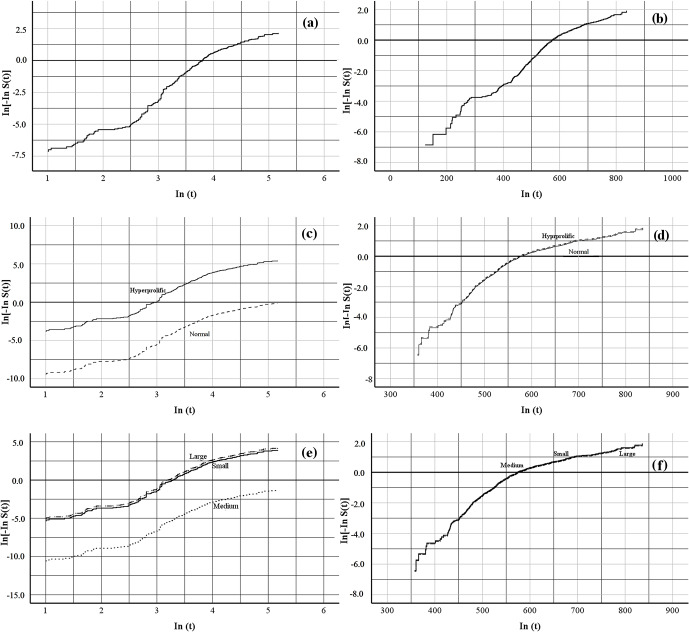
Graphical proof of the Weibull assumption. Linear regression of In[-InS(t)] on In(t) for the length of the productive life of the sow by farrowing number, where panel **(a)** shows the overall productive life, panel **(c)** shows the productive life determined by sow type, and panel **(e)** shows the productive life determined by herd size, and days of life in the herd, where panel **(b)** shows the overall productive life, panel **(d)** shows the productive life determined by sow type, and panel **(f)** shows the productive life determined by herd size, in commercial herds. S(t) denotes the Kaplan–Meier survival function estimates at time t .

To test whether a Weibull distribution correctly fitted the data, the log-rank test of the Kaplan–Meier (nonparametric) estimates of survival curves was plotted against log time. If the Weibull assumption holds, a straight line is obtained. The three analyzed traits (global risk, risk associated with ST, and risk associated with HS) showed linear behavior in both indicators: farrowing number (FN), Y=-9.34 ± 1.16×1.22 ± 0.35×FN ( p<0.0001 ; Fig. 1a, c, e), and days of life in the herd (DLH), Y=-8.92 ± 0.69×0.014 ± 0.001×DLH ( p<0.0001 ; Fig. 1b, d, f).

For multivariate analysis, the Cox proportional hazards model included indicators with a probability within the model of α≤0.05 . The following models were used to determine the risk of removal:

2λ(t)=λ0(t)exp⁡(ST+HS+season+LD+REP+WEI+TPB+PBA+PM),3λ(t)=λ0(t)exp⁡(NPD+TPB+PBA+PM).

Here, λ(t) is the risk function, which represents the risk of a sow being removed after farrowing or days within the herd; $\lambda_{0}(t)$ is the reference hazard function, which is an arbitrary function describing the natural aging process; ST is the time-dependent fixed effect of the sow type and has two levels, normal and hyperprolific sows; HS is the time-dependent fixed effect of herd size and has three levels, small ( <3000 sows), medium (3000–5000 sows), and large ( >5000 sows); season is a time-dependent fixed effect and has four levels, spring, summer, fall, and winter; LD is the time-dependent fixed effect of lactation duration and has two levels, ≤21 and >21 d; REP is the time-dependent fixed effect of the return to estrus percentage; WEI is the time-dependent fixed effect of the weaning–estrus interval; TPB is the time-dependent fixed effect of total piglets born and has six levels (levels considered only for Model 3), 8–9, 10–11, 12–13, 14–15, 16–18, and ≥18 piglets; PBA is the time-dependent fixed effect of piglets born alive and has five levels (levels considered only for Model 3), 6–7, 8–9, 10–11, 12–13, and ≥14 piglets; PM is the time-dependent fixed effect of the percentage of mummies and has four levels (levels considered only for Model 3), 0–10, 11–20, 21–30, and >30 %; and NPD is the time-dependent fixed effect of nonproductive days and has five levels, ≤20 , 21–40, 41–60, 61–80, and >80 d. In Model 3, REP and WEI were omitted, whereas NPD was incorporated. The decision to include NPD was made because this indicator considers both the REP and WEI (Koketsu et al., 2017).

Once the factors affecting the productive longevity of the sows were determined, their behaviors were evaluated. Before the data analysis, the normality of the distribution and homogeneity of the variance for the residuals were determined using PROC UNIVARIATE. A Shapiro–Wilk test was used to determine normality, while a Bartlett test was used to assess homogeneity. Data were analyzed using repeated-measures ANOVA in PROC MIXED (Littell et al., 1998). The sow represents the experimental unit of the model. The effects of sow type (ST); herd size (HS); season; lactation duration (LD); farrowing number (FN); and their main interactions with total piglets born (TPB), piglets born alive (PBA), percentage of mummies (PM), return to estrus percentage (REP), the weaning–estrus interval (WEI), and the nonproductive day (NPD) value were evaluated. The model used was as follows:

4 $$\begin{array}{rl} Y_{\mathrm{ijklmno}}= & \mu +{\text{ST}}_{i}+\text{Sow}(\text{ST}{)}_{j(i)}+{\text{HS}}_{k}+{\text{Season}}_{l}\\ & +{\text{LD}}_{m}+{\text{FN}}_{n}+\text{ST}\times {\text{HS}}_{\mathrm{ik}}\\ & +\text{ST}\times {\text{Season}}_{\mathrm{il}}+\text{ST}\times {\text{LD}}_{\mathrm{im}}\\ & +\text{ST}\times {\text{FN}}_{\mathrm{in}}+\varepsilon_{\mathrm{ijklmno}}. \end{array}$$

Here, $Y_{\mathrm{ijklmno}}$ represents the response variables – TPB, PBA, PM, REP, WEI, and NPD; μ is a common constant of the population; ST ${}_{i}$ is the fixed effect of the i th sow type, where i denotes normal or hyperprolific; Sow(ST) ${}_{j(i)}$ is the random effect of the j th sow nested within the i th sow type, where i denotes normal or hyperprolific; HS ${}_{k}$ is the fixed effect of the k th herd size, where k denotes small, medium, or large; Season ${}_{l}$ is the fixed effect of the l th season, where l denotes spring, summer, fall, or winter; LD ${}_{m}$ is the fixed effect of the m th lactation duration, where m is <21 d or >21 d; FN ${}_{n}$ is the fixed effect of the n th farrowing number, where n is 0,1,2,…,6,≥7 ; ST × HS ${}_{\mathrm{ik}}$ is the fixed effect of the interaction of the i th sow type with the k th herd size; ST × Season ${}_{\mathrm{il}}$ is the fixed effect of the interaction of the i th sow type with the l th season; ST × LD ${}_{\mathrm{im}}$ is the fixed effect of the interaction of the i th sow type with the m th lactation duration; ST × FN ${}_{\mathrm{in}}$ is the fixed effect of the interaction of the i th sow type with the n th farrowing number; and $\varepsilon_{\mathrm{ijklmno}}$ is the random error associated with each observation ( ∼NID=0 , $s_{e}^{2}$ , where NID denotes normality and independence).

The differences between the means were determined using the least-squares means (LsMeans) method, with α≤0.05 . Values are represented as the least-squares mean ± SEM.

## 3 Results

### 3.1 Factors that affect the productive longevity of sows according to survival analysis and Cox regression

**Figure 2:**
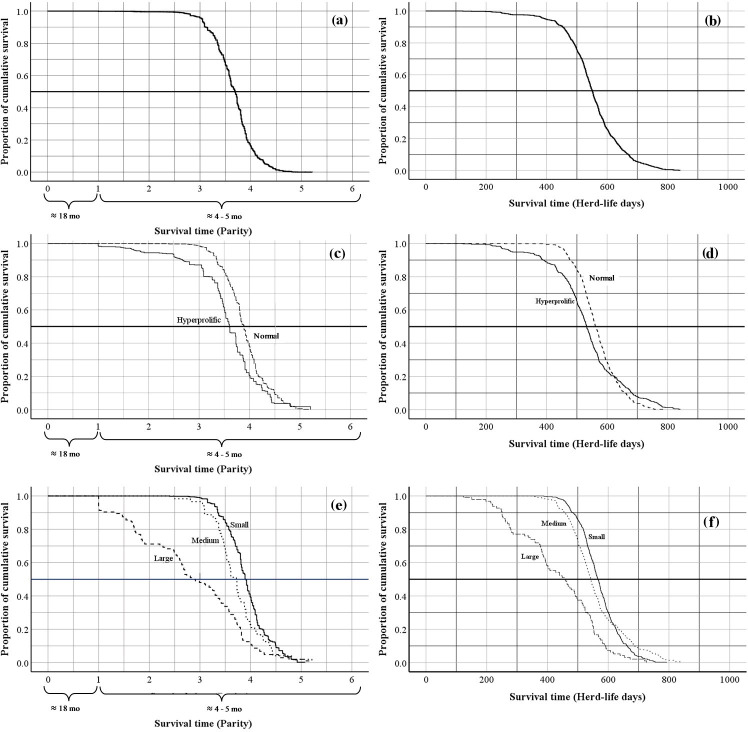
Kaplan–Meier survival curves for overall survival expressed as the farrowing number and days of life of the sow in the herd: **(a, b)** Kaplan–Meier survival curves for the overall productive life of sows; **(c, d)** Kaplan–Meier survival curves for the productive life of sows according to sow type; **(e, f)** Kaplan–Meier survival curves for the productive life of sows according to herd size.

The global average productive life of sows in the SPSs evaluated was 3.7 ± 0.02 farrows in 552.0 ± 2.77 d of life in the herd (Fig. 2a, b). According to the classification of ST, the mean productive life was 3.9 ± 0.02 farrows (in 566.9 ± 3.01 d of life in the herd) for normal sows and 3.5 ± 0.03 farrows (in 532.9 ± 3.91 d of life in the herd) for hyperprolific sows (Fig. 2c, d). Regarding the HS classification, sows belonging to small SPSs had greater productive longevity (3.9 ± 0.02 farrows in 570.3 ± 3.10 d of life in the herd) compared with sows housed in medium and large SPSs (3.7 ± 0.02 and 2.8 ± 0.10 farrows obtained in 559.0 ± 4.89 and 436.0 ± 6.81 d of life in the herd, respectively) (Fig. 2e, f).

**Figure 3:**
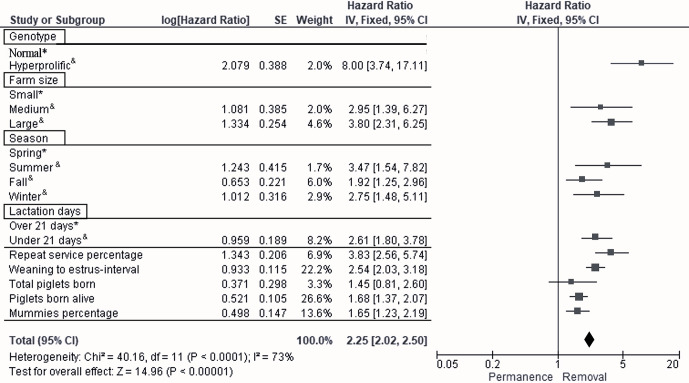
Forest plot based on multivariate hazard ratios (HRs) from Cox regression for the indicators that affect the productive longevity of sows. The squares represent risk ratios. The bars represent 95 % confidence intervals. The square size is proportional to the weights used in the analysis. The diamond represents the overall risk index (middle) with the associated 95 % confidence intervals (side points). ${}^{*}$ reference. ${}^{\&}$ significant p<0.05 .

According to Cox multivariate analysis, the nine time-dependent factors (ST, HS, season, LD, REP, WEI, TPB, PBA, and PM) included in Model 2 were highly significant ( p<0.001 ). The factor with the most important effect in the removal risk analysis was ST, as the removal risk effect was greater for hyperprolific sows: hazard ratio (HR) of 8.0, 95 % confidence interval (CI) of 3.7–17.1 ( p<0.001 ; Fig. 3). The second most important factor with respect to removal risk was the REP (HR of 3.8, 95 % CI of 2.6–5.7, p=0.017 ; Fig. 3), while the third factor was the HS, with large herds showing the greatest effect (HR of 3.8, 95 % CI of 2.3–6.2, p=0.030 ; Fig. 3). The fourth most relevant risk factor for removal was the summer season (HR of 3.5, 95 % CI of 1.5–7.8, p=0.027 ; Fig. 3).

The following factors were established as the fifth to ninth most important factors with respect to the risk of sow removal: LD<21 d (HR of 2.6, 95 % CI of 1.8–3.8, p=0.041 ), WEI (HR of 2.5, 95 % CI of 2.0–3.2, p=0.039 ), PBA (HR of 1.7, 95 % CI of 1.4–2.1, p=0.001 ), PM (HR of 1.6, 95 % CI of 1.2–2.2, p=0.001 ), and TPB (HR of 1.4, 95 % CI of 0.8–2.6, p=0.047 ) (Fig. 3).

**Figure 4:**
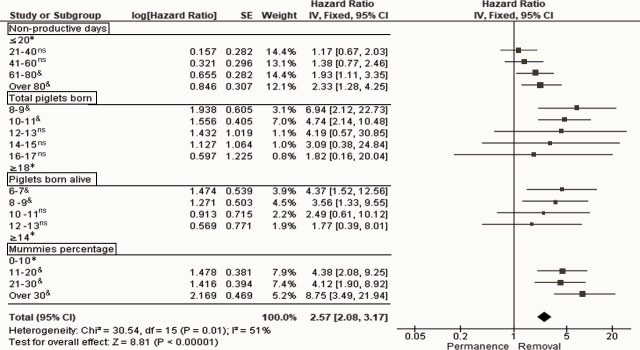
Forest plot based on multivariate hazard ratios (HRs) from Cox regression for the indicators that affect the productive longevity of sows according to different classification levels. The squares represent risk ratios. The bars represent 95 % confidence intervals. The square size is proportional to the weights used in the analysis. The diamond represents the overall risk index (middle) with the associated 95 % confidence intervals (side points). ${}^{*}$ reference. ${}^{\&}$ significant, p<0.05 . ${}^{\text{ns}}$ not significant, p>0.05 .

Following Model 3, the NPD variable was incorporated as an indicator that considered WEI and REP. According to the classification levels of the number of nonproductive days, only the levels of 61–80 and >80 d were significant ( p<0.05 ); these two levels were those that provided the highest risk of sow removal (with regards to NPD): HR of 1.9 (95 % CI of 1.1–3.4) and HR of 2.3 (95 % CI of 1.3–4.2) for the 61–80 and >80 d levels, respectively (Fig. 4).

**Figure 5:**
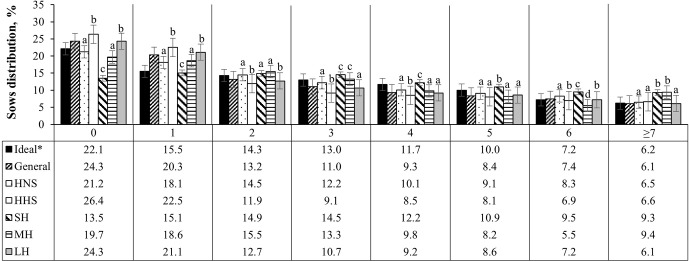
Age structure of sows (farrowing number) in Mexican swine production systems. The abbreviations used are as follows: HNS – herds with normal sows, HHS – herds with hyperprolific sows, SH – small herd, MH – medium herd, and LH – large herd. ${}^{*}$ Ideal farrowing structure to guarantee greater longevity and productive efficiency of the herd (Koketsu, 2007). Different letters (a–d) indicate statistical difference ( p<0.05 ) within the farrowing number.

Regarding TPB, according to the classification levels of this indicator, only the levels of 8–9 (HR of 6.9, 95 % CI of 2.1–22.7) and 10–11 (HR of 4.7, 95 % CI of 2.1–10.5) TPB affected the risk of sow removal (Fig. 4). The same behavior was observed for PBA; however, only the two lower levels ( ≤9 PBA) were considered for the risk of removal of sows: HR of 4.4 (95 % CI of 1.5–12.6) and 3.6 (95 % CI of 1.3–9.5) for the level of 6–7 and 8–9 PBA, respectively (Fig. 4). Regarding the levels at which PM was classified, sows were at risk of removal when the PM was >10 %, with the highest risk of removal when the PM was >30 % (HR of 8.7, 95 % CI of 3.5–21.9) with respect to PM classified as ≤30 % (Fig. 4).

### 3.2 Behavior of the productive indicators that affect the productive longevity of the sow

Following the distribution of the structure of the herd according to FN, it was observed that the herds that have hyperprolific sows and larger herds are those with the highest ( p<0.05 ) percentage of nulliparous sows (26.4 % and 24.3 %, respectively) and first farrows (20.3 % and 21.1 %, respectively), compared with the other classifications of the herds evaluated (Fig. 5). Small herds presented the highest percentage of sows between the third and fifth farrows (37.7 % accumulated percentage) with respect to the other herd classifications; the accumulated percentage of sows between the third and fifth parities was observed to be between 25.7 % and 31.4 % (Fig. 5).

The productive behavior of the sows, according to the TPB, PBA, PM, REP, WEI, and NPD indicators, was evaluated based on the effects of ST, HS, season, LL, FN, and their main interactions (Tables 2, 3). For TPB, an ST effect was observed ( p<0.0001 ), with hyperprolific sows exhibiting the largest litter sizes ( p<0.05 ; Table 2). According to the ST × HS interaction, no effect was observed on TPB ( p=0.0821 ; Table 2). The effect of the ST × season interaction ( p<0.001 ) shows that winter was the season in which the sows, regardless of ST, presented the largest ( p<0.05 ) litter size: 14.7 and 13.8 TPB for hyperprolific and normal sows, respectively. Regarding the interaction between ST × duration of previous lactation, normal sows presented a lower ( p<0.05 ) number of TPB when their previous lactation was <21 d, whereas the reduction in the number of TPB with lactations of <21 d was not significant in hyperprolific sows ( p=0.0634 ; Table 2). Regarding the ST × FN interaction for TPB, the third and fourth farrows had the highest ( p<0.05 ) number of TPB in normal sows, whereas this was found for farrows four and six for hyperprolific sows (Table 2).

**Table 2:** Comparisons between factors for total piglets born, piglets born alive, and percentage of mummies.

	Total piglets born		Piglets born alive		Mummies percentage	
	Normal	Hyperprolific	Normal	Hyperprolific	Normal	Hyperprolific
	13.4 ${}^{1}$ (0.10)	14.3 ${}^{2}$ (0.11)	11.5 ${}^{1}$ (0.12)	12.0 ${}^{2}$ (0.13)	6.9 ${}^{1}$ (0.51)	7.8 ${}^{1}$ (0.55)
Herd size						
Small	13.3 ${}^{\text{a1}}$ (1.11)	13.4 ${}^{\text{a1}}$ (0.59)	11.5 ${}^{\text{a1}}$ (1.22)	11.8 ${}^{\text{a1}}$ (1.56)	6.8 ${}^{\text{a1}}$ (5.05)	4.8 ${}^{\text{a1}}$ (1.45)
Medium	13.5 ${}^{\text{a1}}$ (0.75)	14.2 ${}^{\text{a1}}$ (1.23)	10.8 ${}^{\text{a1}}$ (0.99)	12.1 ${}^{\text{a1}}$ (1.46)	5.1 ${}^{\text{a1}}$ (3.60)	7.8 ${}^{\text{a1}}$ (2.23)
Large	13.7 ${}^{\text{a1}}$ (0.38)	14.1 ${}^{\text{a1}}$ (0.44)	12.2 ${}^{\text{a1}}$ (0.29)	12.0 ${}^{\text{a1}}$ (1.34)	3.5 ${}^{\text{a1}}$ (0.80)	9.5 ${}^{\text{a1}}$ (2.01)
Season						
Spring	13.4 ${}^{\text{a1}}$ (0.21)	14.2 ${}^{\text{a2}}$ (0.18)	11.7 ${}^{\text{a1}}$ (0.24)	11.8 ${}^{\text{a1}}$ (0.21)	6.3 ${}^{\text{a1}}$ (0.98)	8.5 ${}^{\text{a2}}$ (0.87)
Summer	13.4 ${}^{\text{a1}}$ (0.23)	14.3 ${}^{\text{a2}}$ (0.25)	11.8 ${}^{\text{a1}}$ (0.26)	12.1 ${}^{\text{a1}}$ (0.28)	5.2 ${}^{\text{a1}}$ (1.10)	6.1 ${}^{\text{a2}}$ (1.16)
Fall	13.3 ${}^{\text{a1}}$ (0.18)	13.8 ${}^{\text{a2}}$ (0.28)	11.6 ${}^{\text{a1}}$ (0.20)	12.2 ${}^{\text{a2}}$ (0.32)	9.2 ${}^{\text{b1}}$ (0.83)	9.6 ${}^{\text{b2}}$ (1.34)
Winter	13.8 ${}^{\text{b1}}$ (0.24)	14.7 ${}^{\text{b2}}$ (0.24)	11.9 ${}^{\text{a1}}$ (0.27)	12.4 ${}^{\text{a1}}$ (0.27)	5.3 ${}^{\text{a1}}$ (1.12)	6.3 ${}^{\text{a1}}$ (1.14)
Lactation duration						
<21 d	13.0 ${}^{\text{a1}}$ (0.13)	14.1 ${}^{\text{a2}}$ (0.16)	11.6 ${}^{\text{a1}}$ (0.15)	11.7 ${}^{\text{a1}}$ (0.18)	6.2 ${}^{\text{a1}}$ (0.61)	6.6 ${}^{\text{a1}}$ (0.77)
>21 d	13.7 ${}^{\text{b1}}$ (0.18)	14.5 ${}^{\text{a2}}$ (0.16)	11.4 ${}^{\text{a1}}$ (0.21)	12.1 ${}^{\text{a2}}$ (0.19)	8.3 ${}^{\text{a1}}$ (0.88)	9.1 ${}^{\text{a1}}$ (0.78)
Farrowing number						
1	13.1 ${}^{\text{a1}}$ (0.23)	14.1 ${}^{\text{ac2}}$ (0.21)	10.8 ${}^{\text{a1}}$ (0.26)	11.9 ${}^{\text{a2}}$ (0.23)	10.4 ${}^{\text{a1}}$ (1.15)	11.9 ${}^{\text{a1}}$ (1.04)
2	12.6 ${}^{\text{a1}}$ (0.30)	13.4 ${}^{\text{ac2}}$ (0.27)	11.1 ${}^{\text{b1}}$ (0.34)	11.3 ${}^{\text{a1}}$ (0.31)	4.4 ${}^{\text{b1}}$ (1.51)	9.4 ${}^{\text{b2}}$ (1.38)
3	14.3 ${}^{\text{b1}}$ (0.27)	14.1 ${}^{\text{ac1}}$ (0.29)	12.4 ${}^{\text{b1}}$ (0.33)	12.2 ${}^{\text{a1}}$ (0.33)	6.1 ${}^{\text{b1}}$ (1.38)	6.2 ${}^{\text{b1}}$ (1.47)
4	13.8 ${}^{\text{b1}}$ (0.29)	15.0 ${}^{\text{b2}}$ (0.31)	12.1 ${}^{\text{b1}}$ (0.33)	12.7 ${}^{\text{b1}}$ (0.35)	4.5 ${}^{\text{b1}}$ (1.46)	7.2 ${}^{\text{b1}}$ (1.57)
5	13.3 ${}^{\text{a1}}$ (0.25)	14.5 ${}^{\text{a2}}$ (0.31)	11.6 ${}^{\text{b1}}$ (0.28)	12.4 ${}^{\text{a1}}$ (0.35)	6.0 ${}^{\text{b1}}$ (1.25)	6.7 ${}^{\text{b1}}$ (1.57)
6	13.2 ${}^{\text{a1}}$ (0.25)	15.1 ${}^{\text{b2}}$ (0.41)	11.2 ${}^{\text{a1}}$ (0.28)	12.8 ${}^{\text{b2}}$ (0.46)	6.8 ${}^{\text{b1}}$ (1.25)	7.4 ${}^{\text{b1}}$ (2.08)
≥7	12.8 ${}^{\text{a1}}$ (0.26)	13.4 ${}^{\text{c1}}$ (0.32)	10.7 ${}^{\text{a1}}$ (0.29)	11.0 ${}^{\text{c1}}$ (0.37)	7.8 ${}^{\text{b1}}$ (1.31)	7.1 ${}^{\text{b1}}$ (1.63)

${}^{\text{a–c}}$ Different letters indicate statistical differences ( p<0.05 ) within the column for each factor. ${}^{1,2}$ Different numerals indicate statistical differences ( p<0.05 ) within the row for each indicator.

For PBA, the same behavior as that of TPB was observed when evaluating the effect of ST ( p=0.0027 ) and the ST × HS interaction ( p=0.0739 ; Table 3). The effect ( p=0.0019 ) of the ST × season interaction did not show seasonal differences within the ST. Among the ST, only fall presented significant differences ( p<0.05 ; Table 2). According to the ST × duration of the previous lactation interaction, hyperprolific sows presented the highest ( p<0.05 ) number of PBA when lactation was >21 d (Table 2). According to the ST × FN interaction for PBA, normal sows had a higher ( p<0.05 ) number of PBA between the second and fifth farrows, whereas this was found between farrows four and six for hyperprolific sows (Table 2).

Regarding PM, no ST effect ( p=0.2020 ), ST × HS interactions ( p=0.1745 ), or ST × duration of the previous lactation interactions ( p=0.1046 ) were observed. The ST × season interaction determined ( p=0.0024 ) that both STs presented the highest ( p<0.05 ) PM in fall (Table 2). The ST × FN interaction showed ( p=0.0146 ) that first-farrowing sows had the highest ( p<0.05 ) PM in both STs (Table 2).

For REP, no ST effect was observed ( P=0.2831 ). According to the ST × HS interaction ( p=0.0013 ), hyperprolific sows showed higher ( p<0.05 ) REP in medium and large herds (Table 3). The ST × season interaction showed ( p=0.0085 ) that summer was the season in which the highest REP ( p<0.05 ) occurred in both STs (Table 3). Regarding the ST × duration of previous lactation interaction, hyperprolific sows with previous lactation of <21 d presented the highest ( p<0.05 ) REP (18.0 %). The ST × FN interaction indicated ( p=0.0073 ) that sows experiencing their first, sixth, or seventh or higher farrows had the highest REP ( p<0.05 ) in both STs (Table 3).

**Table 3:** Comparisons between factors for return to estrus, weaning–estrus interval, and nonproductive days.

	Return to estrus, %		Weaning–estrus interval, d		Nonproductive days, d	
	Normal	Hyperprolific	Normal	Hyperprolific	Normal	Hyperprolific
	14.2 ${}^{1}$ (0.83)	15.4 ${}^{2}$ (0.75)	7.0 ${}^{1}$ (0.33)	8.1 ${}^{2}$ (0.36)	47.8 ${}^{1}$ (1.21)	37.1 ${}^{2}$ (1.23)
Herd size						
Small	9.0 ${}^{\text{a1}}$ (0.29)	10.5 ${}^{\text{a1}}$ (0.43)	7.1 ${}^{\text{a1}}$ (0.25)	7.4 ${}^{\text{a1}}$ (0.41)	49.1 ${}^{\text{a1}}$ (1.03)	42.4 ${}^{\text{a1}}$ (1.15)
Medium	9.7 ${}^{\text{a1}}$ (0.33)	15.9 ${}^{\text{b2}}$ (0.79)	6.6 ${}^{\text{a1}}$ (0.21)	7.6 ${}^{\text{a1}}$ (0.39)	50.2 ${}^{\text{a1}}$ (3.08)	37.9 ${}^{\text{b2}}$ (0.98)
Large	12.3 ${}^{\text{b1}}$ (0.74)	16.7 ${}^{\text{b2}}$ (2.23)	7.9 ${}^{\text{b1}}$ (0.53)	8.8 ${}^{\text{b2}}$ (0.37)	34.7 ${}^{\text{b1}}$ (1.35)	36.1 ${}^{\text{b2}}$ (1.11)
Season						
Spring	14.1 ${}^{\text{a1}}$ (1.77)	13.6 ${}^{\text{ab}}$ (1.82)	5.9 ${}^{\text{a1}}$ (0.66)	7.7 ${}^{\text{a2}}$ (0.57)	48.8 ${}^{\text{a1}}$ (2.18)	39.3 ${}^{\text{a2}}$ (1.89)
Summer	16.7 ${}^{\text{b1}}$ (2.02)	19.8 ${}^{\text{b2}}$ (1.82)	7.3 ${}^{\text{b1}}$ (0.55)	8.4 ${}^{\text{a2}}$ (0.78)	48.4 ${}^{\text{a1}}$ (2.44)	33.9 ${}^{\text{a2}}$ (2.58)
Fall	14.2 ${}^{\text{a1}}$ (2.41)	14.4 ${}^{\text{a1}}$ (1.53)	6.6 ${}^{\text{a1}}$ (0.72)	8.3 ${}^{\text{a2}}$ (0.89)	52.1 ${}^{\text{a1}}$ (1.83)	37.0 ${}^{\text{a2}}$ (2.96)
Winter	14.5 ${}^{\text{a1}}$ (2.24)	13.8 ${}^{\text{a1}}$ (2.11)	6.4 ${}^{\text{a1}}$ (0.74)	8.4 ${}^{\text{a2}}$ (0.76)	38.7 ${}^{\text{b1}}$ (2.40)	36.1 ${}^{\text{a2}}$ (2.53)
Lactation duration						
<21 d	14.2 ${}^{\text{a1}}$ (1.68)	18.0 ${}^{\text{a2}}$ (1.22)	7.6 ${}^{\text{a1}}$ (0.40)	8.4 ${}^{\text{a2}}$ (0.52)	51.8 ${}^{\text{a1}}$ (1.97)	41.4 ${}^{\text{a2}}$ (1.72)
>21 d	13.3 ${}^{\text{a1}}$ (1.65)	14.1 ${}^{\text{b1}}$ (1.66)	6.8 ${}^{\text{a1}}$ (0.59)	7.8 ${}^{\text{a1}}$ (0.51)	45.9 ${}^{\text{b1}}$ (1.34)	33.7 ${}^{\text{b2}}$ (1.74)
Farrowing number						
1	16.3 ${}^{\text{a1}}$ (1.76)	16.8 ${}^{\text{a1}}$ (1.60)	9.8 ${}^{\text{a1}}$ (0.68)	10.5 ${}^{\text{a1}}$ (0.63)	43.2 ${}^{\text{a1}}$ (1.90)	40.5 ${}^{\text{a1}}$ (1.76)
2	12.8 ${}^{\text{b1}}$ (2.32)	11.4 ${}^{\text{b1}}$ (2.15)	6.9 ${}^{\text{b1}}$ (0.94)	8.4 ${}^{\text{b2}}$ (0.86)	34.8 ${}^{\text{b1}}$ (2.65)	32.0 ${}^{\text{b1}}$ (2.56)
3	13.4 ${}^{\text{b1}}$ (2.17)	12.4 ${}^{\text{b1}}$ (2.27)	5.7 ${}^{\text{b1}}$ (0.86)	7.7 ${}^{\text{b2}}$ (0.91)	37.5 ${}^{\text{b1}}$ (2.42)	29.8 ${}^{\text{b1}}$ (2.42)
4	13.5 ${}^{\text{b1}}$ (2.01)	12.0 ${}^{\text{b1}}$ (5.52)	5.9 ${}^{\text{b1}}$ 80.91)	7.3 ${}^{\text{b2}}$ (0.97)	31.1 ${}^{\text{b1}}$ (2.56)	41.5 ${}^{\text{b2}}$ (2.74)
5	14.5 ${}^{\text{b1}}$ (1.93)	12.4 ${}^{\text{b2}}$ (2.48)	6.5 ${}^{\text{b1}}$ (0.78)	6.9 ${}^{\text{b1}}$ (0.97)	33.4 ${}^{\text{b1}}$ (2.19)	43.9 ${}^{\text{b2}}$ (2.74)
6	16.4 ${}^{\text{a1}}$ (2.33)	14.3 ${}^{\text{a2}}$ (2.44)	6.4 ${}^{\text{b1}}$ (0.78)	5.8 ${}^{\text{b1}}$ (1.30)	52.0 ${}^{\text{c1}}$ (2.19)	48.3 ${}^{\text{c1}}$ (3.63)
≥7	17.8 ${}^{\text{a1}}$ (2.04)	20.2 ${}^{\text{c2}}$ (3.21)	6.2 ${}^{\text{b1}}$ (0.82)	5.7 ${}^{\text{b1}}$ (1.01)	63.2 ${}^{\text{c1}}$ (2.29)	68.4 ${}^{\text{c1}}$ (2.84)

${}^{\text{a–c}}$ Different letters indicate statistical differences ( p<0.05 ) within the column for each factor. ${}^{1,2}$ Different numerals indicate statistical differences ( p<0.05 ) within the row for each indicator.

Regarding WEI, an ST effect was observed ( p=0.0274 ), and hyperprolific sows presented a higher WEI (8.1 d) than normal sows (7.0 d). The ST × HS interaction showed ( p=0.0196 ) that sows present in large herds had a higher WEI ( p<0.05 ) in both STs (Table 3); however, hyperprolific sows had a higher WEI (Table 3). According to the ST × season interaction ( p=0.0352 ), normal sows presented a higher ( p<0.05 ) WEI in summer (7.3 d). In contrast, hyperprolific sows did not show a difference ( p>0.05 ) in WEI according to the season. However, the WEI was higher ( p<0.05 ) in hyperprolific sows in each season than in normal sows (Table 3). Regarding the ST × duration of previous lactation interaction, hyperprolific sows presented a higher WEI (8.4 d) at α=0.0502 when the previous lactation was <21 d (Table 3). Finally, the ST × FN interaction determined ( p<0.0001 ) that first-farrowing sows had the highest WEI in both normal (9.8 d) and hyperprolific (10.5 d) STs.

For NPD, an ST effect was observed ( p<0.0001 ), and hyperprolific sows showed a lower number ( p<0.05 ) of nonproductive days (10.7 d less) than normal sows (Table 3). The ST × HS interaction showed ( p=0.0153 ) that small (regardless of ST) and medium-sized (for normal sows) herds had higher ( p<0.05 ) NPD values (Table 3). According to the effect ( p<0.0001 ) of the ST × season interaction, hyperprolific sows had lower ( p<0.05 ) NPD values during each evaluated season (Table 3). The ST × duration of the previous lactation interaction showed ( p<0.0001 ) that both normal and hyperprolific sows with lactations of >21 d presented a lower ( p<0.05 ) number of nonproductive days (Table 3). Finally, the ST × FN interaction showed ( p<0.0001 ) that the sows of the first, sixth, and seventh or more parities presented the highest ( p<0.05 ) NPD value in both STs (Table 3).

## 4 Discussion

In the current SPSs, it is essential to maximize the reproductive potential of sows to reduce herd production costs and economic inefficiency (Stalder et al., 2004). Therefore, extending the productive life of sows is currently the goal for commercial herds. The productive longevity (PL) of sows is commonly measured by the farrowing number at the time of sow removal (Stalder et al., 2004; Patterson and Foxcroft, 2019; Koketsu and Iida, 2020). Reports exist that the mean parity at the time of sow removal varies from farrowing numbers of 3.3 to 5.6 (Koketsu et al., 1999, 2020; Lucia et al., 2000; Rodriguez-Zas et al., 2003; Engblom et al., 2007). Regarding what was observed in the evaluated herds in this work, the average farrowing number at the time of removal was 3.7 (Fig. 2a). However, it has been reported (Rodriguez-Zas et al., 2003; Koketsu and Iida, 2020) that sow removal based on the farrowing number is not an accurate way to monitor the PL of sows, as it does not consider the number of days that the sow remains in the herd, which can vary between herds for sows of the same farrowing number. Regarding this indicator, it was discovered that sows in the evaluated herds have a general average herd lifetime of 552.0 d (Fig. 2b). This result is in the lower range of what has previously been reported (between 467 and 969 d) in herds from the US, EU, and Japan (Rodriguez-Zas et al., 2003; Saito et al., 2010; Koketsu et al., 2020).

The importance of guaranteeing greater PL lies in the increased productivity of sows between the third and fifth parities (Rodriguez-Zas et al., 2003; Gruhot et al., 2017). The lower PL values of the sows and the distribution of the herd, according to the FN, were oriented to the left, with a higher percentage of gilts and primiparous sows. According to Koketsu (2007), the ideal farrowing structure to guarantee greater herd longevity and productive efficiency should consider 22.1 % nulliparous sows, 29.8 % primiparous sows (first and second farrows), 34.7 % multiparous sows (third to fifth farrows), and 13.4 % old sows (sixth farrows or above). Pregnant nulliparous females and first-farrowing sows had lower reproductive performance than sows between the third and fifth farrows, which included lower fertility, lower PBA, and higher WEI, as observed in the evaluated herds (Tables 2, 3).

This can be explained by the immature endocrine system of these animals and their feed intake during lactation (Weldon et al., 1994; Mosnier et al., 2010). This last factor (low feed intake during the first lactation) is reflected in the second litter syndrome, which favors the production of fewer piglets (Table 2) because gonadotropin secretion decreases, leading to restricted growth of follicles in the ovaries (Hoving et al., 2011; Sell-Kubiak et al., 2021). Older sows show lower reproductive performance than sows between the third and fifth farrows (Tables 2, 3); this is due to the fact that ovulation and fertility rates decrease, and older sows thus tend to have higher embryo mortality or pregnancy loss as well as more stillborn piglets (Segura et al., 2020). In addition, older individuals and gilts have a higher risk of miscarriage than multiparous sows (Bertoldo et al., 2012), which generates a greater number of nonproductive days (Table 3).

According to the results of this work, it was observed that the evaluated herds generally present a higher percentage of nulliparous (24.3 %) and primiparous (33.5 %) sows. Among the factors evaluated that affect sow longevity, genotype (prolific or hyperprolific sows) and HS were the first and third factors, respectively, that put sows at greater risk ( p<0.05 ) of removal from the herd. These indicators altered the farrowing structure, resulting in a higher percentage of nulliparous and primiparous sows associated with lower productive longevity of the sow, 3.5 (532.9 d of life in the herd) and 2.8 (436.0 d of life in the herd) farrows for hyperprolific sows and large herds, respectively.

According to the results regarding the ST, it has been reported that administrative management, different management practices of each system, and the environment can potentiate or block the genetic potential of sows (Ortiz, 2019; He et al., 2019; Patterson and Foxcroft, 2019); this is observed in the behavior of reproductive and productive indicators, such as REP, WEI, PBA, and TPB, which are the second, sixth, seventh, and ninth most important factors affecting the removal of sows from the evaluated herds, respectively (Fig. 3).

With the current genotypes of hyperprolific sows genetically selected for lean meat production and greater feed efficiency during the growing–finishing stage, the physiology, productivity, and feed efficiency of future breeders remain unaffected (Foxcroft, 2012). Another aspect of modern hyperprolific sows is an increase in litter size. Over the last 20 years, the productive potential of these sows has reached an average of 19.6 piglets per litter (Tokach et al., 2019), which favors greater energy demand in sows. During lactation, milk production is prioritized over most other metabolic processes; therefore, most lactating sows undergo catabolic processes (Pedersen et al., 2019). Nutrient requirements for milk production often exceed the dietary intake of sows, mobilizing body fat and proteins for milk production (Kim et al., 2001). This is reflected in a loss of body weight, and weight loss during lactation compromises the development of ovarian follicles (growth and quality) owing to the irregular production of metabolic intermediaries, such as luteinizing hormone (Knox, 2015). The development of these compromised follicles results in a lower ovulation rate, lower embryonic and fetal survival, and a higher REP (Mejia-Guadarrama et al., 2002).

Roughly 20 to 40 years ago, alterations in follicular development in sows were practically nonexistent, and this was associated with a longer duration of lactation ( ≥28 d) and lower reproductive intensity; therefore, the effects on ovulation rate and embryo survival were minimal (Xue et al., 1993; Leenhouwers et al., 2011). This behavior was observed when evaluating the duration of lactation (the fifth factor that influenced the removal of sows in the evaluated herds). Sows that presented previous a lactation of >21 d showed a greater litter size and PBA as well as lowered REP and WEI in the subsequent reproductive cycle, with hyperprolific sows being the most benefited (Tables 2, 3). The fact that sows' reproductive and productive indicators improve with an increase in the duration of lactation is associated with a decrease in milk production at the end of the third week of lactation (Hansen et al., 2012) and the greater animal feed intake during that period (Knauer and Hostetler, 2013), which causes the sows to enter a catabolic state during the last lactation period. Follicle development and litter size are not compromised by the catabolic state, as is the case with lactations of <21 d (Leenhouwers et al., 2011; Kemp and Soede, 2012).

As established in the previous paragraph, a high feed intake capacity and optimal feed composition are crucial variables, particularly for hyperprolific sows. Feed intake is controlled by several factors, including feed composition, body condition, farrowing, ambient temperature, genotype, and milk production (Eissen et al., 2000). Considering the effect of environmental temperature on feed intake, an negative effect of increased air temperature on feed intake during lactation has been reported (Bjerg et al., 2020): feed intake was decreased by between 230 and 270 $g\;d^{-1}$ at average temperatures of between 25 and 27 °C . However, Quiniou and Noblet (1999) established that this decrease in feed intake could begin before the air temperature reaches 25 °C . According to the analyzed herds, it was observed that, with respect to season (the third factor that affects the risk of sows being discarded in the evaluated herds), summer had the greatest risk of sow removal, which may be associated with low feed intake during lactation and its subsequent effect on the reproductive and productive indicators, namely, PBA, WEI, and REP (Tables 2, 3). Moreover, it is important to consider the seasonal effect as a factor that influences the behavior of the indicators that affect the productive longevity of the sow, as a large part of commercial SPSs, mainly in developing countries, is an open to the environment; therefore, managers do not have full control over the regulation of environmental temperatures (Nava et al., 2009; INIFAP, 2018).

Regarding HS, existing studies have reported contrasting results. Koketsu et al. (2020) showed that Spanish herds had a higher number of PBA (0.3 piglets) during the first farrowing period when the herd increased from 180 to 1300 sows. This was associated with the premise that large herds experience genetic improvement in less time, have a better health status, and have better production systems (with state-of-the-art infrastructure) compared with small herds (Koketsu, 2000; Knox et al., 2013). However, it has also been reported (Rodriguez-Zas et al., 2003; Koketsu et al., 2020) that the longevity of sows is lower in larger herds – an aspect observed in Mexican herds (Fig. 1e, f). In large herds, the age at first mating increases, which is reflected in decreased productive longevity, prolificacy, fertility, and sow efficiency compared with small or medium herd sizes (Babot et al., 2003; Koketsu et al., 2020).

Regarding the results observed in the herds evaluated in this work, this lower sow efficiency was reflected in a higher REP and WEI (Table 3), with the REP being the second most important factor with respect to discarding sows from the herds analyzed, based on the results of the Cox proportional hazards analysis (Fig. 3). Additionally, it is important to highlight that, although SPSs (mainly large herds) count on technological investment by acquiring hyperprolific sows and improving infrastructure, this strategy does not guarantee productive success. The incorporation of new technologies requires an internal analysis of the specific SPS in question, as the implemented technologies are generally foreign and were developed to solve problems specific to the place where they were developed; therefore, to maximize their implementation, intellectual capital and learning were created (Ortiz, 2019).

With respect to PM, the eighth most important indicator affecting the removal of sows from the evaluated herds, a mummification rate of between 1.5 % and 3.5 % has been reported (Borges et al., 2005) as normal. This level is lower than that observed in this study (Table 3). Fetal mummification is associated with infectious diseases, FN, litter size, uterine capacity, environmental temperature, and mycotoxins (Mengeling et al., 2000; Borges et al., 2005). Regarding the high general percentage of mummified piglets in the evaluated herds, PRRSV (porcine reproductive and respiratory syndrome virus) is endemic in the evaluated area and was the main infectious agent increasing the number of mummified piglets (López et al., 2015). The trend of a higher percentage of mummification in hyperprolific sows is associated with the larger litter size and uterine capacity of this type of sow (le Cozler et al., 2002). However, with respect to season, fall presents the highest PM values; this rise in the PM coincides with the addition of grain (corn or sorghum) to feed at the end of the season, which is characterized by an increase in the concentration of mycotoxins at that time (van der Lende and van Rens, 2003).

Finally, observational studies have limitations that do not exist in controlled experiments. For example, herd health, nutrition, and husbandry practices may not be well controlled in observational studies. In addition, a few trademark data may have been recorded incorrectly. Similarly, multiple observations per sow were not independent of the observation units. However, even with these limitations, the analysis of SPS data using appropriate exclusion criteria and multilevel statistical models can provide practical and easily applicable field-level information on production problems that are difficult to investigate via controlled experiments.

## 5 Conclusions

With respect to the risk of sow removal from the herd, there are two factors inherent to animals at the farm level: ordinary factors and performance factors. Regarding the herds evaluated, this study identified both ordinary and inherent factors as the highest risk factors for sow removal from the herd, including genotype, environmental factors (season), and duration of lactation. Hyperprolific sows, sows farrowing in summer, and sows lactating for less than 21 d had a higher risk of involuntary removal. Among the performance factors that put sows in herds at greater risk of removal are the return to estrus percentage, the weaning–estrus interval, the number nonproductive days ( >60 d), the litter size ( <12 piglets), the number of piglets born alive ( <10 piglets), and the percentage of mummies ( >10 %). At the herd level, herd size was identified as a risk factor for sow removal: the larger the herd, the greater the risk of sow removal from the herd. Therefore, the productive longevity of sows within a herd is determined by the type of sow and their association with environmental disturbances, including climatic factors (artificial climate control), management practices (human resources), and economic resources (size and infrastructure).

## Data Availability

The original data used in this study are available from the corresponding author upon request.
